# Pathological Physiology

**Published:** 1868-01-15

**Authors:** 


### PATHOLOGICAL PHYSIOLOGY.

### THE CONTINUOUS VENOUS MURMURS IN THE NECK.

*Clinical Lectures delivered at the Hospital "La Charitè," by M. Monneret, Professor of Clinical Medicine to the Faculty of Medicine, Paris.*

TRANSLATED EXPRESSLY FOR THE JOURNAL, BY WALTER HAY, M.D., CHICAGO.

(*Continued from page* 13.)

I COME now to the most important consideration of all in the question which we are now studying; I refer to the composition of the blood. I made numerous experiments upon this subject in 1847.

It is manifest that, of two liquids, advancing with equal rapidity, flowing at the same inclination, starting from the same height, and subjected to the same pressure, the one which produces the most intense sound is that which wets the most thoroughly the walls of the tube enclosing it. If a mixture of water and alcohol be used, the most intense sound and the most beautiful modulations are produced. The same effects are obtained with a solution of table salt or of carbonate of soda. If, on the contrary, the experiment is made with viscous, thick or ropy liquids, which flow silently, and whose molecules adhere together, scarcely any sound is obtained. Such is the result of experiments performed with pure milk, with a mixture of milk and water, with oil, or with a decoction of marshmallow. I have been enabled to use these liquids in large quantity, and to experiment, in a complete manner, in the public hospitals.

*Mercury*, which does not moisten, rolls also without sound; but it must be added that *Mercury* exists under peculiar conditions. According to physicists, it does not possess the properties of liquids, and should be put completely out of the question. I have never produced vibrations with *Mercury*, whatever may have been the height of its fall. All those liquids which moisten thoroughly, flow more rapidly than those which moisten imperfectly.

It is always the velocity of the liquid which produces the continuous sounds and the swells, continuous and intermittent murmurs. Such is the conclusion necessarily reached, whether the liquid be permitted to flow, or be forced mechanically; and even, I do not hesitate to say — although this word has offended some very sensitive persons — with a syringe.

Others may jest about experiments, which are somewhat rude, it is true; but when these experiments reproduce with perfect exactness the phenomena announced by such as Savart and Cagnard-Latour, it is then time to be serious, and to assert physical laws, which are superior to these feeble attacks.

The experiments which M. Parrot thought it necessary to attribute to M. Chauveau, I had already made in 1847.

In 1833, Laharpe (de Lausanne) investigated the influence of liquids upon the production of sounds, and arrived at results identical with those I have exhibited to you.

M. Parrot has therefore misquoted. If I notice this error, it is simply in order to establish a fact which is often observed in the incomplete historical researches, which each one presents in his own manner.

I have never thought of claiming priority in investigations which have been for a long time already public property.

After what has gone before, I hope, gentlemen, that you can not cherish any doubt about the influence of the composition of liquids upon the production of sounds. If you apply what I have said to the sanguineous liquid, you perceive that, when this liquid is modified in such a manner as to become more fluid, to wet the walls of the vessel, and to flow more easily, the vascular murmur of the neck appears, it is on the right that you find it; you know to what anatomical causes this is due in part. Two conditions suffice to produce it; the one is constant — this is the aponeurotic arrangement; the other is variable — this is the composition of the blood. Nature, in order to arrive at her ends, has no need to multiply mechanisms; it is sufficient to add to them some modifications. It belongs to the physician to apply vital laws to physical objects.

Thus, the material arrangement does not suffice to originate a murmur, notwithstanding the cause is always persistent. A still further modification is necessary, which should cause the phenomena to reappear every time the appearance of a vascular sound, of the nature of those we are studying, is established; and it can be determined that the blood is altered in its composition. It is useless to analyze them; others have done it before us, and with such precision that we can not refute their evidence.

What I say of the venous murmurs, I say also of the vibratile thrill; for they can not be separated, the one from the other. They appear together — 1st, when the blood is diminished in quantity; 2nd, when it has lost a part of its most essential element — that is to say, when there is a diminution of the globules. The globules seem indeed to render the blood more viscous, and to give it the property of rolling silently in the vessels. Its velocity then is not so great, and it becomes impossible to produce a sound in the veins, in the normal condition; but if the quantity of the globules falls from 127 to 100, and even still lower to 90, 80, or even to 75, the blood acquires a great quantity of serum, and becomes more aqueous; the diminution of globules is thus compensated by the augmentation of the water, as Andral and Gavarret have demonstrated. In their analyses, the serum is perceived to ascend to 790, 800, or 900, in the 1000. It is to this greater fluidity, that is due the venous murmur. When it is carried to its maximum, it is a true tidal murmur that is heard. It is at times so intense that it has occurred to me, on three different occasions, to hear it at a distance. Imagine how powerful must be a sonorous vibration, in order to pass from the liquid where it is at first produced, into the air, then from this fluid to the ear, with which it is put in unison by the intermediation of the air.

To conclude, then, I assert that each time a bellows murmur is heard, it may be certainly diagnosed; with Andral and Gavarret, that the blood has no longer its normal composition; and that its globules have fallen to 80 or 90. I recognize in this diagnostic sign, one of the most beautiful applications of physical and chemical facts to practical medicine.

The blood, modified as to its quantity, ought to produce likewise the same result. Previous to the labors of Andral and Gavarret, this diminution in the quantity of the blood in anæmia was admitted; it is only since that period, that a diminution in the number of globules merely has been recognized. In spite of the numerous experiments of physiologists, we do not know the quantity of liquid blood contained in the organism; but by considering the smallness of the veins in the anæmia, the decolorized condition of the blood obtained by puncture, and the inconsiderable quantity which is found after death in the sick person who dies, it becomes necessary to ask if there be not a real diminution in the mass of the blood. I am inclined to believe that it is so, in spite of the high authority of Andral. This is not an easy question; for, in this case, the blood would flow with a velocity so much greater, as it would, at the same time, less abundant and less dense.

The bellows-murmur may announce also the diminution of a third element; this element is the albumen, diminishes in a manner protopathic or deuteropathic. When the alteration of the solids forbids the formation of this element, or entails its immediate elimination, the murmur of anæmia becomes apparent. This alteration of the solids exists, for example, in a disease of the kidneys, which removes from the blood its albumen, or of every other organ whose derangements react upon the general health, and forbid the formation of albumen. Moreover, the albumen is perceived to diminish, and to produce the same effect, when blood is lost in large quantity, as in important wounds, in severe parturitions. There is in all these cases, a disappearance of a large portion of the essential elements of the blood, the albumen and the globules; the blood then moistens more thoroughly the walls of the vessels, as in deglobulization. There is perceived, also, according to the old opinions, the diminution of the other elements of the blood, the fibrin and the fatty matters, under the influence of imperfect nutrition, as Bright, Christison, Andral, and others, have perceived. And this is not all — there is still a loss of the inorganic principles, the different salts, the protein bodies, or extractives; in such cases, the murmur becomes the most intense.

If, now, we examine the first causes of anæmia and its murmur, we shall enter upon the study of disease which I designate under the name of inanition. By this is to be understood, not only loss of nutritive matter by reason of deficiency of aliment; there is inanition when the nutrition is impaired by any cause whatever — for example, the altered nervous system prevents the stomach assimilable, for a still stronger reason when the stomach is itself altered, when it is the seat of a cancer or of an ulceration which impedes its functions.

In these cases, there is inanition because the sick person can not recruit his blood with the requisite quantity of necessary material.

Anæmia is, moreover, the result of many other diseases.

It is sufficient that the liver be diseased, in order that the elements essential to the organism be not renewed; the globules then diminish. Biliary calculi, cirrhosis, whatever it be, primitive or consequetive — acepalocysts, even, which at first do not appear able to affect it much; in a word, all that which disturbs the functions of organs, produces anæmia. Amongst such invalids, even when this malady is not suspected, it is necessary to seek for the vibratile thrill and the venous murmur.

You will find them with such intensity that you can not be doubtful. It might be said, for the rest, that every slow and chronic lesion of an organ manifests its development by anæmia.

The spleen is often the source of it, without any doubt. Among leucæmic subjects, who have in their blood two, or even four, white to one red globule, the venous murmurs appear from the commencement, and put the observer upon the track of the malady, before he has even had recourse to percussion. You see, then, how important is the examination of these murmurs; and I have not yet said all, for it is not in general disease, which has for its result a rapid alteration of the blood, that this alteration of the blood becomes often one of the most essential elements of diagnosis.

The alteration of the blood is a constitutional effect of different poisons. In order that the liquid should remain in its natural state, it is necessary that the different principles which flow into it, should not be susceptible of alteration.

It is thus that a subject who does not appear sick, and who comes to consult us about a trifling discomfort, is already profoundly affected. You determine in his case, anæmia; you interrogate him with care, and he informs you that he is a house-painter; you conclude that his blood is altered, perhaps that digestion is not perfectly performed, perhaps that absorption has introduced a poison into his system — the contact of the lead has sufficed to modify his blood, to disturb all the functions of the organism, some feebly, others to a high degree; and, without making an analysis of the blood, you affirm the fact with certainty. Subsequently, if there is need of it, an analysis of the blood will demonstrate to you that the globules have diminished in quantity. I cite lead; but any other poison happens to produce the same effect, by attacking the blood globules, and either by destroying them or by preventing their formation.

*Pellagra*, *Ergotism*, which are poisons frequently mortal, alter the blood, and develop anæmia rapidly, in the same manner. Is it necessary to cite to you all the maladies with which you are abundantly familiar, and which are followed by the same effect? I will only specify the rheumatic and gouty diatheses, etc., cancer, and tubercle. It is sufficient that a subject should have the diathesis, in order that, a long time — perhaps ten years — before the least external manifestation appears, he becomes anæmic. A young girl is a prey to the tubercular diathesis, and she may have still the appearance of health; she is only chlorotic. Two years, four years pass, and you commence to suspect a condition more grave. You auscultate with care, and you find the signs of nascent tuberculization; the origin of this evil is with the parents; the diathesis was in its embryonic state, and only exhibited itself by the alteration of the blood.

It is certain that the opinions which we express will meet with determined opposition, because they are associated with thoughts of sadness, and because they touch the heart in its tenderest emotions; but this is a conviction, and I am compelled to declare that the subject has been struck fatally, from its birth, by the diathesis.

These sad thoughts have the merit of making the observer more attentive, and of permitting him to commence the treatment at the moment when he can still rely upon the efficacy of hygiene. Such is its true progress. If a diathesis can determine anæmia in an individual, from the beginning, what will it be when it shall have broken out in all its vigor, and when it shall have developed itself with all its manifestations? The invalid will then become anæmic with a promptness such as will approximate to the state of an individual who has lost blood, or in whom one of the larger veins has been divided. Two or three days of an acute disease will have accomplished a result at which a chronic disease would have arrived only after a very long time.

Now, there is a sure method of ascertaining the effects produced by this malady upon the entire organism; and I am acquainted with none of them which can determine the venous murmurs so rapidly and so surely as one of these general diseases.

One word, now, upon the subject of the bellows murmur which is met with in an arterio-venous aneurism. This aneurism gives origin, indeed, as surgeons have determined, to the two phenomena which we have studied — to the continuous bellows murmur, duplicated with an intermittent blowing, and to the vibratile thrill. The explanations furnished by authors, of the origin of these murmurs, are scarcely in harmony with the principles adopted by physicists.

When it has been said that this murmur is due to the vibration of the perforated membrane intermediate to the two vessels, and that the passage of the blood from the artery into the veins, suffices to explain the continuous murmur — a greater rapidity of the current during the diastole, explaining also the swell — there has been given an explanation of the phenomenon which is not correct. This explanation is a complete contradiction in physics.

In the memoir which I presented to the Surgical Society in 1852, I sought to demonstrate the true causes of this murmur. There can be, in the transit through the vessel, but two simultaneous sounds, or a single one, a continuous and an intermittent sound.

There can be a continuous sound, only, in a vessel through which the blood passes with sufficient rapidity, and in a continuous manner. This blood ought, as the experiments of physicists has demonstrated, to accomplish a certain distance, to run through a certain space, in order to generate a vibration. These conditions exist in the veins near the heart; the flow of blood into the arteries is remittent; every one knows that it is subjected to two influences — the systole of the heart, and the tension of the artery; but the murmur is heard only during the cardiac systole, the continuous flow not being sufficiently strong to produce a second: the first one is therefore intermittent.

Thus the blood flows silently during the arterial systole; it flows with a certain murmur during the arterial diastole, because then the range is sufficient. Now, Scarpa has demonstrated that arterial blood can not enter into a vein without arterializing it. It results from this that the vein becomes rigid, and that the blood flows in this vessel of a larger diameter, with thicker walls, and always distended, in a continuous manner. It is the artery which gives out the intermittent sound, and the vein which affords the continuous; these two vessels are found united in the arterio-venous aneurism, and hence the reason why there is heard a continuous-remittent bellows murmur. The intermittent sound is engrafted upon the continuous, as the artery is upon the arterialized vein altered in its walls.

This is clear, and it is not necessary to go to seek in the walls of membranes, vibrations which do not exist, as M. Broca has done, who appears to me to have announced a false theory of the arterio-venous murmur.

I will not give you a longer description of the vibratile thrill, and of the continuous venous murmurs. You have heard these phenomena, and you have studied them in the sick. Moreover, I must dwell upon the operative proceedings which enable them to be recognized. It is not, however, always easy to perceive them clearly, especially if one has not taken care beforehand to place the head of the subject in a suitable position.

Remember the arrangement of the aponeuroses of the neck, and the intimate relations which they sustain to the veins of the region. The costo-pericardiac ligament, as has been noticed by MM. Lannelonge and Ledentio, maintains these vessels in contact with the superior bony rim of the thorax; it prevents its sinking, and preserves a free and easy passage to the blood which runs through them; it subserves a purely passive purpose. The omo-hyoid aponeurosis, or middle layer, on the contrary, an active part; it is extended, under the influence of the contraction of the scapulo-hyoid — or better still, by the extension of the head; and under the influence of this tension, the vascular walls are kept apart, and thus the circulation is accelerated.

The action of the superficial aponeurosis, relative to the external jugular, is similar. You should, then, in the first place, endeavor to stretch these membranes; and you will affect it easily by turning the head upon the opposite side, and elevating it sufficiently to elongate the muscles of the neck. The subject should be placed in the horizontal position; the erect is bad. If it is desired to observe the vibratile thrill, or the blowing, in all their intensity, the pulp of the finger or the stethoscope should be placed immediately above the right clavicle. Take care, also, in the different examinations made, that the stethoscope is exactly applied. Only very light pressure should be made, and the pulp of the finger should just graze the skin a little, in order to perceive the vibratile thrill. If the compression is too strong, no result will be attained. I will not dwell upon all the details upon which I might still enter. Remember, however, that the vascular signs, even when sought with care, are sometimes difficult to recognize, and are very mobile. They constitute, at the least, two valuable symptoms, which permit the diagnosis of a general disease in its incipiency, and enable it to be studied in its rudimentary state.

It is by similar evidences that we shall be at some time enabled to attain to a recognition of the alterations in the blood, the existence of diathesis, and also to arrest in time the progress of disease by an appropriate hygiene.

We could thus suspect, at an early period, the existence of those organic modifications which, at a later date, we are powerless to arrest.

We can not cure diatheses — we can oppose to them only palliatives. Some day, perhaps, recognized early, we shall be enabled to suspend their evolution.

